# Vaccination Coverage Among Children in Kindergarten — United States, 2012–13 School Year

**Published:** 2013-08-02

**Authors:** Ranee Seither, Lauren Shaw, Cynthia L. Knighton, Stacie M. Greby, Shannon Stokley

**Affiliations:** Immunization Svcs Div, National Center for Immunization and Respiratory Diseases, CDC

State and local school vaccination requirements are implemented to maintain high vaccination coverage and minimize the risk from vaccine preventable diseases (1). To assess school vaccination coverage and exemptions, CDC annually analyzes school vaccination coverage data from federally funded immunization programs. These awardees include 50 states and the District of Columbia (DC), five cities, and eight U.S.-affiliated jurisdictions.* This report summarizes vaccination coverage from 48 states and DC and exemption rates from 49 states and DC for children entering kindergarten for the 2012–13 school year. Forty-eight states and DC reported vaccination coverage, with medians of 94.5% for 2 doses of measles, mumps, and rubella (MMR) vaccine; 95.1% for local requirements for diphtheria, tetanus toxoid, and acellular pertussis (DTaP) vaccination; and 93.8% for 2 doses of varicella vaccine among awardees with a 2-dose requirement. Forty-nine states and DC reported exemption rates, with the median total of 1.8%. Although school entry coverage for most awardees was at or near national *Healthy People 2020* targets of maintaining 95% vaccination coverage levels for 2 doses of MMR vaccine, 4 doses of DTaP† vaccine, and 2 doses of varicella vaccine (2), low vaccination and high exemption levels can cluster within communities, increasing the risk for disease. Reports to CDC are aggregated at the state level; however, local reporting of school vaccination coverage might be accessible by awardees. These local-level data can be used to create evidence-based health communication strategies to help parents understand the risks for vaccine-preventable diseases and the benefits of vaccinations to the health of their children and other kindergarteners.

Vaccination coverage among children entering kindergarten is assessed annually by awardees. Each school year, the health department, school nurse, or other school personnel assess the vaccination and exemption status of a census or sample of kindergarteners enrolled in public and private schools to determine vaccination coverage, as defined by state and local school requirements established to protect children from vaccine-preventable diseases. Among the 50 states and DC, 43 awardees used an immunization information system (IIS) as at least one source of data for some of their school assessment. To collect data, 33 awardees used a census of kindergarteners; 11 a sample of schools, kindergarteners, or both; two a voluntary response of schools; and five a mix of methods. Results of the school-level assessments are reported to the health department. Aggregated data are reported to CDC for public and private schools. Data for homeschooled students were not reported to CDC. All estimates of coverage and exemption were weighted based on each awardee’s response rates and sampling methodology, unless otherwise noted. Of the 50 states and DC, 12 awardees met CDC standards for school assessment methods in 2012–13.§

Kindergarteners were considered up-to-date for each vaccination if they had received all of the doses required for school entry in their jurisdiction. School entry requirements varied by awardee: all reporting awardees required 2 doses of MMR vaccine; for DTaP vaccine, two awardees required 3 doses, 35 required 4 doses, and 20 required 5 doses; and for varicella vaccine, 13 required 1 dose, 41 required 2 doses, and three did not require varicella vaccination.

The types of exemptions allowed varied by awardee. All reporting awardees allowed medical exemptions, 46 allowed religious exemptions, 18 allowed philosophic exemptions, and two (Mississippi and West Virginia) did not allow exemptions for religious or philosophic reasons. Medical, religious, and philosophic exemptions were reported as the percentage of kindergarteners with each type of exemption. Total exemptions were reported as the percentage of kindergarteners with any exemption.

Overall, among the 48 states and DC that reported 2012–13 school vaccination coverage, median 2-dose MMR vaccination coverage was 94.5% (range: 85.7% in Colorado to ≥99.9% in Mississippi); 20 reported coverage ≥95% (Table 1). Median DTaP vaccination coverage was 95.1% (range: 82.9% in Colorado and Arkansas to ≥99.9% in Mississippi); 25 reported coverage ≥95%. Median 2-dose varicella vaccination coverage among the 36 states and DC requiring and reporting 2 doses was 93.8% (range: 84.6% in Colorado to ≥99.9% in Mississippi); 14 reported coverage ≥95%.

An estimated 91,453 exemptions were reported among a total estimated population of 4,242,558 kindergarteners. Overall, among the 49 states and DC that reported 2012–13 school vaccination exemptions, the percentage of kindergarteners with an exemption was <1% for nine awardees and >4% for 11 awardees (range: <0.1% in Mississippi to 6.5% in Oregon), with a median of 1.8% (Figure; Table 2). The largest increases in total exemptions between 2011–12 and 2012–13 were reported by Georgia and West Virginia, each with an increase of 1.0 percentage point; four states reported decreases of >1.0 percentage points (range: −1.3 in Colorado to −1.6 in New Mexico). Where reported separately, the median medical exemption level was 0.3% (range: <0.1% in five states [Arkansas, Minnesota, Mississippi, North Dakota, and Virginia] to 1.6% in Alaska). Where allowed and reported separately, the median nonmedical exemption level was 1.5% (range: 0.2% in New Mexico to 6.4% in Oregon).

## Editorial Note

Kindergarten vaccination coverage for most reporting awardees remained high and exemption levels remained stable for the 2012–13 school year compared with the 2011–12 school year. Although high levels of vaccination coverage at the awardee level is reassuring, vaccine-preventable disease outbreaks can still occur among clusters of unvaccinated persons at local levels in schools and communities (3,4). Vaccination exemptions have been shown to cluster geographically (5,6). If exemption levels are high in a school or community, the number of unvaccinated kindergarteners might be sufficient to permit transmission of vaccine-preventable diseases, if introduced. Assessing and reporting school vaccination coverage at the local level is critical for state education and health departments to protect kindergarteners and the community from vaccine-preventable diseases. Among the 50 states and DC, a total of 11 awardees reported local-level data online, ensuring that local level data are widely available.¶ These local-level data can be used by health departments and schools to develop health communication strategies based on the specific vaccine-preventable disease risk at a local school caused by low vaccination coverage or high exemption levels.

An exemption does not necessarily imply a child was not vaccinated. More than 99% of the 2006–2007 birth cohorts who became kindergarteners in 2012–13 received at least one vaccine (7). Additionally, in some areas, a parent or guardian may complete the required exemption paperwork if the kindergartener’s vaccination history cannot be easily documented at school enrollment (8,9). Less stringent exemption standards have been associated with higher numbers of exemptions (8,9).

What is already known on this topic?

Vaccine-preventable diseases continue to be transmitted despite high levels of vaccination at the national and state levels. School vaccination assessment can help local health officials determine the risk for vaccine-preventable disease transmission at the local level.

What is added by this report?

For the 2012–13 school year, median vaccination coverage in the 48 states and the District of Columbia continued to be high, with medians of 94.5% for measles, mumps, rubella; 95.1% for diphtheria, tetanus toxoid, and acellular pertussis; and 93.8% for varicella vaccines. The level of exemptions remained low overall, with a median of 1.8%, and four awardees saw decreases of >1 percentage point for children with exemptions in the 2012–13 school year.

What are the implications for public health practice?

High vaccination coverage levels at the national and state levels might mask clustering of unvaccinated children at local levels where vaccine-preventable diseases might be transmitted. Health departments and school systems can use local-level school vaccination assessment data to identify schools with low vaccination coverage and high exemption levels. This local-level evidence can be used to develop local-level health communication campaigns and other strategies to ensure parents understand vaccine-preventable disease risks and vaccination benefits.

School vaccination coverage assessment is a local-level data reporting system required as part of state or local level school vaccination requirements. CDC supports the use of standards to improve the ability to use the school vaccination coverage data to reliably monitor local vaccination coverage, including appropriate sampling methods, data collection by trained staff, and validation. One way to improve the quality of vaccination coverage reporting is to link school vaccination assessment systems to an IIS. In 2011, 45 of 51 awardees allowed schools to obtain data from their IIS, of which 43 awardees reported using the IIS capacity to complete their school vaccination assessment reporting and 20 were able to generate reports for school vaccination coverage (10). Allowing school vaccination assessment systems to link to awardee IIS data can help ensure provider-reported vaccinations are reported to the schools, minimizing the reporting burden on busy parents and schools, and might help health and education departments identify local areas with low levels of vaccination coverage.

The findings in this report are subject to at least two limitations. First, these data are cross-sectional, collected at a single point in the school year. Vaccination and exemption status reflected the child’s status at the time of assessment. Some children might have been in the process of receiving required vaccines and final vaccination or exemption status might have changed after the survey was completed. Reports might or might not be updated as a child obtained the required vaccines or claimed an exemption later in the school year. Vaccination and exemption status might not have been reported for every child. Second, data collection and methodology varied by awardee and even by school year for the same awardee. Methods and times for data collection differed, as did requirements for vaccinations and exemptions.

School vaccination assessments provide valuable information for state and local immunization programs about vaccination coverage, exemptions, and clustering of unvaccinated kindergarteners in schools, where the potential for disease transmission is higher. Data at the state level alone can mask areas of undervaccination. Health departments can access and use local school vaccination assessment data to target areas for vaccination interventions during outbreaks of vaccine-preventable disease. This information also can be used by health departments and schools to develop evidence-based health communication strategies and other interventions that protect kindergarteners and the community against vaccine-preventable diseases.

## Figures and Tables

**FIGURE:**
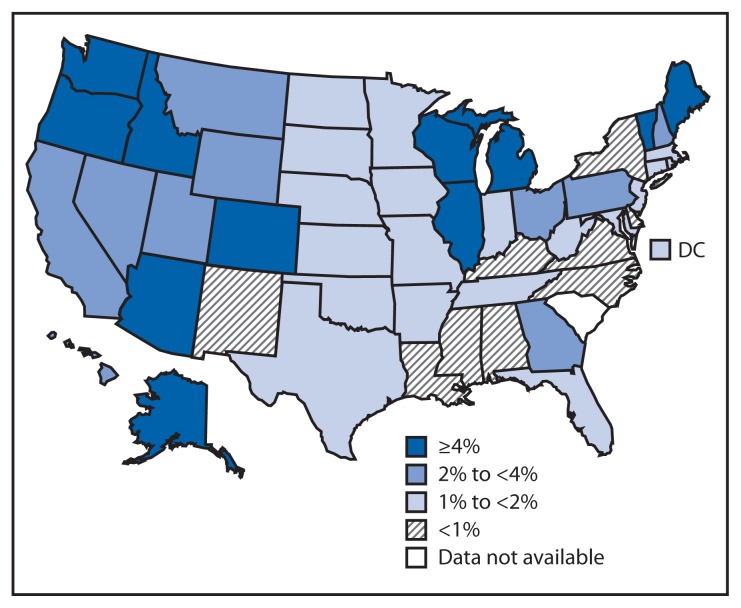
Estimated percentage of children enrolled in kindergarten who have been exempted from receiving one or more vaccines* — United States, 2012–13 school year * Exemptions might not reflect a child’s vaccination status. Children with an exemption who did not receive any vaccines are indistinguishable from those who have an exemption but are up-to-date for one or more vaccines.

**TABLE 1:** Estimated vaccination coverage* among children enrolled in kindergarten, by state/area, type of survey conducted, and selected vaccines — United States, 2012–13 school year

State/Area	Estimated kindergarten population	Total surveyed	Proportion surveyed (%)	Type of survey conducted†	MMR§ (%)	DTaP/DT¶ (%)	Varicella	

							1 dose (%)	2 doses (%)
Alabama**	72,929	**72,929**	(100.0)	Census	(92.8)	(92.8)	(91.9)	NReq
Alaska	10,319	**10,092**	(97.8)	Voluntary response	—††	—††	—	—††
Arizona	90,054	**87,909**	(97.6)	Census	(94.5)	(94.6)	(96.8)	NReq
Arkansas	43,212	**41,602**	(96.3)	Census (public), Voluntary response (private)	(85.9)	(82.9)	—	(84.8)
California**	535,523	**530,418**	(99.0)	Census (public), Voluntary response (private)	(92.7)	(92.5)	(95.6)	NReq
Colorado	70,657	**350**	(0.5)	Simple random	(85.7)	(82.9)	—	(84.6)
Connecticut	41,604	**41,604**	(100.0)	Census	(97.1)	(97.2)	—	(96.8)
Delaware	11,997	**934**	(7.8)	2-Stage cluster	(≥95.7)	(≥95.7)	—	(≥95.7)
District of Columbia	7,842	**7,842**	(100.0)	Census	(91.8)	(91.6)	—	(91.5)
Florida	234,628	**234,628**	(100.0)	Census	(≥92.1)§§	(≥92.1)§§	—	(≥92.1)§§
Georgia	142,732	**3,388**	(2.4)	2-Stage cluster	(≥95.1)	(≥95.1)	—	(≥95.1)
Hawaii**	20,104	**1,339**	(6.7)	2-Stage cluster	(97.3)	(98.0)	(99.3)	NReq
Idaho	23,888	**23,888**	(100.0)	Census	(88.4)	(88.3)	—	(86.6)
Illinois	166,884	**166,884**	(100.0)	Voluntary response	(95.5)	(94.6)	(96.6)	NReq
Indiana	86,983	**72,532**	(83.4)	Census	(95.4)	(90.1)	—	(93.9)
Iowa	41,701	**41,663**	(99.9)	Census	(≥90.5)	(≥90.5)	—	(≥90.5)
Kansas	40,738	**12,943**	(31.8)	Mixed design (public), Census (private)	(90.0)	(88.9)	—	(88.4)
Kentucky**	58,466	**56,846**	(97.2)	Census (public), Voluntary response (private)	(92.4)	(94.1)	—	(89.5)
Louisiana	68,874	**68,874**	(100.0)	Census	(96.6)	(98.3)	—	(96.5)
Maine**	14,313	**13,533**	(94.6)	Census	(91.3)	(95.2)	(95.1)	NReq
Maryland**	75,007	**63,212**	(84.3)	Census	(98.2)	(99.4)	(99.6)	NReq
Massachusetts**	79,661	**77,767**	(97.6)	Census	(94.8)	(93.1)	—	(93.6)
Michigan	124,662	**124,662**	(100.0)	Census	(94.4)	(95.1)	—	(92.9)
Minnesota**	73,310	**73,310**	(100.0)	Census	(96.3)	(96.7)	—	(95.9)
Mississippi	46,595	**46,595**	(100.0)	Census	(≥99.9)	(≥99.9)	—	(≥99.9)
Missouri	78,416	**4,634**	(5.9)	2-Stage cluster	(96.7)	(96.5)	—	(96.1)
Montana	12,516	**11,990**	(95.8)	Census	(94.8)	(94.5)	—	NReq
Nebraska**	24,999	**24,153**	(96.6)	Census	(96.0)	(95.5)	—	(94.7)
Nevada	36,070	**1,561**	(4.3)	2-Stage cluster	(93.6)	(96.6)	—	(92.2)
New Hampshire	12,943	**12,839**	(99.2)	Census	—††	—††	—	—††
New Jersey	122,516	**118,447**	(96.7)	Census	(≥97.0)	(≥97.0)	—	(≥97.0)
New Mexico	29,279	**971**	(3.3)	2-Stage cluster	(93.9)	(96.6)	—	(94.0)
New York**	239,484	**239,484**	(100.0)	Census	(96.6)	(98.4)	(98.4)	NReq
North Carolina	130,612	**127,150**	(97.3)	Census	(97.3)	(97.2)	(98.0)	NReq
North Dakota	9,503	**466**	(4.9)	2-Stage cluster	(89.9)	(89.0)	—	(88.5)
Ohio	163,687	**142,170**	(86.9)	Census	(96.3)	(96.3)	—	(95.7)
Oklahoma	56,943	**54,381**	(95.5)	Census (public), Voluntary response (private)	(90.5)	(90.2)	(92.8)	NReq
Oregon	47,102	**47,102**	(100.0)	Census	(93.5)	(93.4)	(94.5)	NReq
Pennsylvania	151,364	**147,582**	(97.5)	Census	(87.0)	(90.7)	—	(85.7)
Rhode Island**	12,552	**9,445**	(75.2)	Census	(94.2)	(95.2)	—	(94.2)
South Carolina	61,799	**11,039**	(17.9)	1-Stage cluster	(≥90.9)	(≥90.9)	—	(≥90.9)
South Dakota	12,468	**12,468**	(100.0)	Census	(97.9)	(97.7)	—	(96.0)
Tennessee	85,801	**83,188**	(97.0)	Census	(≥94.5)	(≥94.5)	—	(≥94.5)
Texas	414,688	**400,510**	(96.6)	Census	(97.5)	(97.3)	—	(97.2)
Houston	34,407	**2,447**	(7.1)	2-Stage cluster (private)	(95.8)	(96.4)	—	(96.2)
Utah	54,605	**54,605**	(100.0)	Census	(96.3)	(97.8)	(99.6)	NReq
Vermont	6,792	**6,792**	(100.0)	Census	(92.8)	(92.6)	—	(90.1)
Virginia	104,826	**4,399**	(4.2)	2-Stage cluster	(93.1)	(98.5)	—	(91.2)
Washington	87,773	**83,082**	(94.7)	Census	(91.7)	(91.9)¶¶	—	(90.3)
West Virginia	22,588	**19,549**	(86.5)	Census	(96.3)	(96.3)	—	(96.2)
Wisconsin	72,416	**1,337**	(1.8)	2-Stage cluster	(92.8)	(96.1)	—	(91.1)
Wyoming	8,133	**8,133**	(100.0)	Census (public)	(97.5)	(97.4)***	—	(97.0)
*Median* †††					(*94.5*)	(*95.1*)		(*93.8*)
American Samoa	NA	**NA**	NA	Not conducted				
Guam	2,835	**1,054**	(37.1)	2-Stage cluster	(89.8)	(91.2)	—	NReq
Marshall Islands	NA	**NA**	NA	Not conducted				
Micronesia	NA	**NA**	NA	Not conducted				
N. Mariana Islands	847	**847**	(100.0)	Census	(54.7)	(92.8)	—	(54.7)
Palau	471	**471**	(100.0)	Census (public)	(76.2)	(84.5)	—	NReq
Puerto Rico**	39,106	**979**	(2.5)	2-Stage cluster	(85.2)	(81.9)	—	(88.7)
U.S. Virgin Islands	1,498	**851**	(56.8)	2-Stage cluster	(92.3)	(78.9)	—	(81.9)

**Abbreviations:** MMR = measles, mumps, and rubella vaccine; DTaP/DT = diphtheria and tetanus toxoids (DT) and acellular pertussis vaccine; NA = not available; NReq = not required for school entry.

* Estimates are adjusted for nonresponse and weighted for sampling where appropriate, except where complete data were unavailable. Percentages for Delaware, Georgia, and Puerto Rico are approximations. Estimates for South Carolina and Colorado were provided by the awardee. Estimates based on a completed vaccine series (i.e., not antigen-specific) are designated by use of the ≥ symbol.

† Sample designs varied by state/area: census = all schools (public and private) and all children within schools were included in the assessment; simple random = a simple random sample design was used; mixed design = a census was conducted among public schools, and a random sample of children within the schools were selected; 1-stage or 2-stage cluster sample = schools were randomly selected, and all children in the selected schools were assessed (1-stage) or a random sample of children within the schools were selected (2-stage); voluntary response = a census among those schools that sent in assessment data.

§ Most awardees require 2 doses; California, Illinois, New York, and Oregon require 2 doses of measles, 1 dose of mumps, and 1 dose of rubella.

¶ DTaP vaccination coverage might include some DTP (diphtheria and tetanus toxoids and pertussis) or DT vaccinations if administered in another country or vaccination provider continued to use after 2000. Most awardees require 4 doses of DTaP/DT vaccine; 5 doses are required for school entry in Colorado, District of Columbia, Hawaii, Idaho, Iowa, Kansas, Massachusetts, Minnesota, Mississippi, North Carolina, Oregon, Rhode Island, Texas (including Houston), Vermont, Washington, Wyoming, Northern Mariana Islands, and Puerto Rico; 3 doses are required by Nebraska and New York; 4 doses of DT and 2 doses of pertussis vaccine are required by the U.S. Virgin Islands. Pertussis vaccine is not required in Pennsylvania; the estimate for Pennsylvania represents DT only.

** Awardee counts the vaccine doses received regardless of Advisory Committee on Immunization Practices recommended age and time interval; vaccination coverage rates might be higher than those recommended.

†† Vaccination coverage and exemption levels were reported together. Vaccination coverage estimates could not be provided separately for this report.

§§ Does not include nondistrict-specific, virtual, and college laboratory schools, or private schools with less than 10 students.

¶¶ For DT only; coverage for pertussis was 92.4%.

*** Diphtheria coverage was 97.3%; tetanus and pertussis were 97.4%.

††† The median is the center of the estimates in the distribution. The median does not include Alaska, New Hampshire, Houston, Guam, the Commonwealth of the Northern Mariana Islands, Palau, Puerto Rico, and the U.S. Virgin Islands.

**TABLE 2:** Weighted number and percentage* of children enrolled in kindergarten with a reported exemption to vaccination, by state/area and type of exemption — United States, 2012–13 school year

	Medical exemptions†		Nonmedical exemptions†				Total exemptions†			
			
State/Area	No.	(%)	Religious no.	Philosophic no.	Total no.	(%)	Total no.	2012–13 (%)	2011–12 (%)	Percentage point difference
Alabama	64	(0.1)	414	—§	**414**	(0.6)	**501**	(0.7)	(0.6)	0.1
Alaska	162	(1.6)	415	—§	**415**	(4.0)	**564**	(5.6)	(7.0)	−1.4
Arizona	315	(0.3)	—¶	—¶	**3,475**	(3.9)	**3,790**	(4.2)	(3.7)	0.5
Arkansas	17	(0.0)	99	342	**441**	(1.0)	**458**	(1.1)	(0.9)	0.2
California	923	(0.2)	—**	14,921	**14,921**	(2.8)	**15,845**	(3.0)	(2.6)	0.4
Colorado	191	(0.3)	0	2,678	**2,678**	(4.0)	**2,869**	(4.3)	(5.6)	−1.3
Connecticut	124	(0.3)	601	—§	**601**	(1.4)	**725**	(1.7)	(1.3)	0.4
Delaware	2	(0.2)	5	—§	**5**	(0.5)	**7**	(0.7)	(0.6)	0.1
District of Columbia	101	(1.3)	27	—§	**27**	(0.3)	**128**	(1.6)	(2.1)	−0.5
Florida	905	(0.4)	3,281	—§	**3,281**	(1.4)	**4,186**	(1.8)	(1.5)	0.3
Georgia	4	(0.1)	73	—§	**73**	(2.2)	**77**	(2.3)	(1.3)	1.0
Hawaii††	17	(0.3)	138	—§	**138**	(2.2)	**156**	(2.5)	(3.9)	−1.4
Idaho	90	(0.4)	171	1,138	**1,309**	(5.5)	**1,399**	(5.9)	(5.4)	0.5
Illinois	2,017	(1.2)	8,082	—§	**8,082**	(4.8)	**10,099**	(6.1)	(5.5)	0.6
Indiana	326	(0.4)	804	—§	**804**	(0.9)	**1,129**	(1.3)	(1.2)	0.1
Iowa	188	(0.5)	500	—§	**500**	(1.2)	**688**	(1.7)	(1.5)	0.2
Kansas	118	(0.3)	363	—§	**363**	(0.9)	**436**	(1.1)	(1.3)	−0.2
Kentucky	128	(0.2)	286	—§	**286**	(0.5)	**414**	(0.7)	(0.6)	0.1
Louisiana	109	(0.2)	27	322	**349**	(0.5)	**458**	(0.7)	(0.8)	−0.1
Maine	61	(0.4)	17	541	**559**	(3.9)	**620**	(4.3)	(3.9)	0.4
Maryland	237	(0.3)	494	—§	**494**	(0.7)	**731**	(1.0)	(0.9)	0.1
Massachusetts	373	(0.5)	843	—§	**843**	(1.1)	**1,216**	(1.5)	(1.4)	0.1
Michigan	699	(0.6)	1,086	5,540	**6,626**	(5.3)	**7,325**	(5.9)	(5.5)	0.4
Minnesota	32	(0.0)	—¶	—¶	**1,149**	(1.6)	**1,181**	(1.6)	(1.6)	0.0
Mississippi	23	(0.0)	—**	—§			**23**	(0.0)	(0.0)	0.0
Missouri	377	(0.3)	1,665	—§	**1,665**	(1.5)	**2,042**	(1.8)	(2.4)	−0.6
Montana	49	(0.4)	380	—§	**380**	(3.0)	**439**	(3.5)	(3.0)	0.5
Nebraska	149	(0.6)	269	—§	**269**	(1.1)	**418**	(1.7)	(1.5)	0.2
Nevada	85	(0.7)	224	—§	**224**	(1.8)	**309**	(2.5)	(1.8)	0.7
New Hampshire	30	(0.2)	298	—§	**298**	(2.3)	**328**	(2.5)	(2.2)	0.3
New Jersey	261	(0.2)	1,458	—§	**1,458**	(1.2)	**1,719**	(1.4)	(1.3)	0.1
New Mexico	20	(0.2)	27	—§	**27**	(0.2)	**47**	(0.4)	(2.0)	−1.6
New York††	331	(0.1)	1,335	—§	**1,335**	(0.6)	**1,666**	(0.7)	(0.7)	0.0
North Carolina††	162	(0.1)	871	—§	**871**	(0.7)	**1,032**	(0.8)	(0.8)	0.0
North Dakota	0	(0.0)	6	123	**130**	(1.8)	**130**	(1.8)	(1.0)	0.8
Ohio	650	(0.4)	—¶	—¶	**2,665**	(1.6)	**3,315**	(2.0)	(1.5)	0.5
Oklahoma	66	(0.1)	179	493	**672**	(1.2)	**738**	(1.3)	(1.1)	0.2
Oregon	72	(0.2)	3,010	—§	**3,010**	(6.4)	**3,010**	(6.4)	(5.9)	0.5
Pennsylvania	643	(0.4)	—¶	—¶	**2,339**	(1.5)	**2,982**	(2.0)	(1.8)	0.2
Rhode Island	45	(0.4)	94	—§	**94**	(0.8)	**139**	(1.1)	(1.0)	0.1
South Carolina	NA				**NA**		**NA**		(1.1)	
South Dakota	41	(0.3)	182	—§	**182**	(1.5)	**223**	(1.8)	(1.2)	0.6
Tennessee	162	(0.2)	905	—§	**905**	(1.1)	**1,066**	(1.2)	(0.7)	0.5
Texas	2,112	(0.5)	—¶	—¶	**4,936**	(1.2)	**7,048**	(1.7)	(1.5)	0.2
Houston	NA		NA	NA	**NA**		**22**	(0.9)	(0.1)	0.8
Utah	83	(0.2)	6	2,010	**2,016**	(3.7)	**2,099**	(3.8)	(3.8)	0.0
Vermont	30	(0.4)	14	371	**385**	(5.7)	**415**	(6.1)	(5.7)	0.4
Virginia	48	(0.0)	427	—§	**427**	(0.4)	**474**	(0.5)	(1.0)	−0.5
Washington††	1,092	(1.2)	274	2,774	**3,048**	(3.5)	**4,077**	(4.6)	(4.7)	−0.1
West Virginia	262	(1.2)	—**	—§			**262**	(1.2)	(0.2)	1.0
Wisconsin	472	(0.5)	276	3,631	**3,907**	(4.0)	**4,380**	(4.5)	(4.5)	0.0
Wyoming	28	(0.3)	155	—§	**155**	(1.9)	**183**	(2.3)	NA	NA
*Median* §§		(*0.3*)				(*1.5*)		(*1.8*)	(*1.5*)	*0.3*
American Samoa	NA				**NA**		**NA**			
Guam	0	(0.0)	0	—§	**0**	(0.0)	**0**	(0.0)	(0.0)	0.0
Marshall Islands	NA				**NA**		**NA**			
Micronesia	NA				**NA**		**NA**			
N. Mariana Islands	1	(0.1)	0	—§	**0**	(0.0)	**1**	(0.1)	(0.0)	0.1
Palau	3	(0.6)	0	0	**0**	(0.0)	**3**	(0.6)	(1.3)	−0.7
Puerto Rico	0	(0.0)	0	—§	**0**	(0.0)	**0**	(0.0)	(0.0)	0.0
U.S. Virgin Islands	0	(0.0)	7	—§	**7**	(0.6)	**7**	(0.6)	(0.5)	0.1

**Abbreviation:** NA = not available (i.e., not tracked or not reported to CDC).

* Estimates are adjusted for nonresponse and weighted for sampling where appropriate, except where complete data were unavailable. Percentages for Delaware, Georgia, and Puerto Rico are approximations. Estimates for South Carolina and Colorado were provided by the awardee. Arkansas and Kansas conduct a census of exemptions.

† Medical and nonmedical exemptions might not be mutually exclusive. Some children might have both medical and nonmedical exemptions. Total exemptions are the number of children with an exemption. Temporary exemptions are included in the total for Alabama, Hawaii, New York, North Carolina, South Carolina, and Washington.

§ Exemptions because of philosophic reasons are not allowed.

¶ Religious and philosophic exemptions are not reported separately.

** Exemptions because of religious reasons are not allowed.

†† Includes both temporary and permanent medical exemptions.

§§ The median is the center of the estimates in the distribution. The median does not include South Carolina, Houston, Guam, the Commonwealth of the Northern Mariana Islands, Palau, Puerto Rico, and the U.S. Virgin Islands.

## Footnotes

* Vaccination coverage and exemption data were reported to CDC by Guam, the Commonwealth of the Northern Mariana Islands, Palau, Puerto Rico, and the U.S. Virgin Islands. No data were reported by American Samoa, the Marshall Islands, and the Federated States of Micronesia. Data from these U.S.-affiliated jurisdictions were excluded from the national analysis.

† The *Healthy People 2020* DTaP vaccination coverage target (IID-10.1) is based on 4 doses of DTaP vaccine. This report describes compliance with state regulations of 3, 4, or 5 doses of DTaP vaccine. Of the 51 awardees, only Nebraska, New York, and Pennsylvania report <4 doses of DTaP vaccine.

§ CDC standards included use of a census or random sample to assess public and private schools or students, assessment using number of doses recommended by the Advisory Committee on Immunization Practices, assessment of vaccination status before December 31, collection of data by health department personnel or school nurses, validation of data collected by school administrative staff, and vaccination documentation from a health-care provider.

¶ Information available, by state, at the following websites: Arizona, http://www.azdhs.gov/phs/immunization/statistics-reports.htm; California, http://www.cdph.ca.gov/programs/immunize/pages/immunizationlevels.aspx; Florida, http://www.doh.state.fl.us/disease_ctrl/immune/resources/surveys/state-surveys.html; Illinois, http://www.isbe.state.il.us/research/htmls/immunization.htm#immu; Iowa, http://www.idph.state.ia.us/immtb/immunization.aspx?prog=imm&pg=auditsl; Kansas, http://www.kdheks.gov/immunize/kindergarten_coverage.htm; New Jersey, http://www.state.nj.us/health/cd/imm_status_report_2012.shtml; Texas, http://www.dshs.state.tx.us/immunize/coverage/schools.shtm; Utah, http://health.utah.gov/immu/statistics/utah%20statistics/immunization%20coverage%20levels/index.html; Vermont, http://healthvermont.gov/hc/imm/ImmSurv.aspx; Washington, http://www.doh.wa.gov/dataandstatisticalreports/schoolimmunization/datareports.aspx.
